# Bacterial profile of high-touch surfaces, leftover drugs and antiseptics together with their antimicrobial susceptibility patterns at University of Gondar Comprehensive Specialized Hospital, Northwest Ethiopia

**DOI:** 10.1186/s12866-021-02378-w

**Published:** 2021-11-08

**Authors:** Atsedewoyn Firesbhat, Abiye Tigabu, Birhanemeskel Tegene, Baye Gelaw

**Affiliations:** 1 Unit of Biomedical Sciences, College of Veterinary Medicine and Animal Sciences, University of Gondar, P O. box: 196, Gondar, Ethiopia; 2 Department of Medical Microbiology, School of Biomedical and Laboratory Sciences, College of Medicine and Health Sciences, University of Gondar, P O. box: 196, Gondar, Ethiopia; 3 Department of Medical Microbiology, St. Paul’s Hospital Millennium Medical College, P. O. box 1271, Addis Abeba, Ethiopia

**Keywords:** Bacterial profile, Antiseptics, High touch surfaces, Leftover drugs

## Abstract

**Background:**

The hospital environment serves as a source of nosocomial infections, which pose a major therapeutic challenge. Although many bacteria species are common in hospital environments, their distribution, frequency, and antimicrobial susceptibility pattern from high-touch surfaces, leftover drugs, and antiseptics in different wards remain largely unknown. Hence, the aim of this study was to assess the magnitude and frequency of bacterial contaminants and their antimicrobial susceptibility patterns.

**Methods:**

A total of 384 samples were collected from five selected wards and processed according to standard bacteriological procedures. Samples were collected from high-touch surface using swabs and inoculated on Blood agar, MacConkey agar, Chocolate agar and Mannitol salt agar plates, and incubated at 37 °C for 24 h. On the other hand, the leftover drugs and 80% ethanol samples were collected using sterile cotton swab immersed in sterile tryptone soy broth then inoculated on culture medias and incubated at 37 °C for 24 h. Identification of bacteria species was done using the morphological characteristics, Gram stain, and biochemical tests while antimicrobial susceptibility tests were done using modified Kirby-Bauer disk diffusion technique following the Clinical Laboratory Standards Institute 2021guidelines.

**Results:**

Among the 384 samples processed, 102 (26.6%) were culture positive and a total of 114 bacterial isolates were identified. Gram-positive bacterial isolates were predominant, 64.9%, while Gram-negatives were 35.1%. The most frequently isolated bacteria were coagulase negative *Staphylococci* (38.6%) followed by *S. aureus* (13.2%) and *P. aeruginosa* (11.4%). On the other hand, the proportion of bacteria isolated from surgical ward, post-natal ward, orthopedic ward, trauma ward, and neonatal intensive care unit ward were 24.6, 21, 20.2, 18.4,15.8%, respectively. Sinks were mainly contaminated with *Klebsiella* species (81.8%) and *A. baumannii* (55.6%), while *A. baumannii* (22.2%) was the most contaminant for 80% ethanol. Gram-positive bacteria had significantly high resistance levels to penicillin (67.6%), cotrimoxazole (67.8%), and cefepime (80%). On the other hand, Gram-negative bacteria revealed the highest resistance levels to tetracycline (82.4%), amoxicillin-clavulanic acid (76.5%), cefepime (66.7%), ceftazidime (67.5%), and piperacillin (92.3%). Moreover, the proportion of multidrug resistant bacteria isolates was 44.7%.

**Conclusions:**

Data of the present study showed that coagulase negative *Staphylococci* was the dominant bacterial isolates followed by *S. aureus* and *P. aeruginosa*. The proportion of multi-drug resistant bacteria isolates was relatively high. Therefore, appropriate infection prevention and control measures should be implemented.

## Background

Nosocomial infections are infections acquired during hospitalization, which are important causes of morbidity and mortality in hospitals throughout the world [1, 2]. Environmental surfaces in health care facilities can be a reservoir for bacteria and serve as a source of nosocomial infections (NIs). Environmental contamination contributes to the transmission of bacteria when health care workers contaminate their hands or gloves by touching contaminated objects, or when patients come into direct contact with contaminated surfaces [3]. Bacteria can be transferred to health care workers and patients [4]. The hand contact surfaces of the hospital environments are the main sources of many bacterial isolates such as *S. aureus, E. coli*, *Enterococcus*, *Streptococcus*, *Acinetobacter*, *Salmonella*, *Shigella*, *Klebsiella*, *Proteus*, and *Pseudomonas* species [5].

High-touch surfaces are frequently contacted surfaces by health care workers, patients, and visitors, which may be a reservoir for nosocomial pathogens and a source for transmission of healthcare-associated pathogens, which has led to multiple outbreaks of healthcare-acquired infections [6, 7]. Leftover drug is the medicine which remains after the consumer has used it. Sterile preparations must be done to control contamination by bacteria and other microbes. During the medication process, both the leftover drugs and the 80% alcohol can be contaminated. Contamination by bacterial isolates is also reported on high touch surfaces. In addition, hands of healthcare workers in particular and the hospital environment in general have been linked to contamination and serious infections and outbreaks [8, 9]. Multidrug-resistant bacteria defined as non-susceptibility to at least one agent in three or more antimicrobial classes. Despite the widespread availability of antibiotics, multidrug-resistant (MDR) bacterial isolates remain a worldwide therapeutic problem [10].

The burden of NIs is much higher in developing countries due to poor ventilation systems, high dusting, overcrowded settings, spread through sneezing and coughing, high movement of personnel [11]. The major contributing factors for increased nosocomial infection rates are overuse of antimicrobials, long-term stay in health care facilities, failure of infection control procedures in many hospitals, and a high number of immune-compromised patients [12]. The increased use of antimicrobial agents, advancement of life-saving medical practices (invasive procedures), and poor infection prevention practices are associated with the ever-increase of hospital-acquired infections in developing countries [13].

Preparation of parenteral medication in hospitals is a complex process with a risk of microbial contamination during reconstitution of the medicines in the clinical area before administration to the patient [14]. The contamination rate of parenteral medication in hospital environments is between 1.09–20.70 and 0.00%–0.66%, respectively [15]. Antiseptics are commonly used antimicrobial agents in health care settings that can kill, inhibit, or reduce the number of microorganisms on the hands of health care workers, the skin of the patients, and achieve surgical hand antiseptics before invasive medical procedures. The human skin has a wide variety of microorganisms that may provide a protective mechanism but can also serve as a source of infection [16, 17]. Antiseptic preparation by unskilled personnel in a contaminated environment, use of unsterilized containers, and prolonged use can lead to microbial contamination, which may contribute to infection and death [18–21].

According to the World Health Organization 2019 NIs fact sheet report, one hundred million patients were affected each year globally and the prevalence of NIs in developed and low- and middle-income countries were estimated between 3.5–12% and 5.7–19.1%, respectively [22]. The emergence of multidrug-resistant bacterial strains in the hospital environment particularly in developing countries is an increasing problem which is an obstacle for management of NIs [23, 24]. The magnitude of multidrug-resistance among the bacterial isolates is becoming critical, and approximately 60% of the NIs involve antimicrobial-resistant bacteria [25–32]. Although there are some study reports on bacterial contamination of the hospital environments in Ethiopia, there is a paucity of information about the bacterial profile contaminating the high touch surfaces, leftover drugs and 80% ethanol together with the drug susceptibility pattern of the bacterial isolates. Therefore, this study was designed to assess the proportion and antimicrobial susceptibility patterns of bacterial isolates from high-touch surfaces, leftover drugs, and 80% ethanol at University of Gondar Comprehensive Specialized Hospital (UoGCSH), Ethiopia.

## Methods

### Study area

The study was conducted at UoGCSH, which is located in Gondar city, Ethiopia. It is one of the largest teaching hospital in the Amhara regional state providing surgical, medical, pediatric, gynecologic, obstetric, oncologic, and ophthalmologic services for more than 7 million people coming from Amhara, Tigray, and Benishangul Gumuz regions. It is a multidisciplinary specialized hospital with 700 inpatient beds and consists of an operating room, intensive care units, fistula center, different wards, and outpatient departments.

### Study design and period

A hospital-based cross-sectional study was conducted at UoGCSH from the 1st of December 2020 to 20th March 2021. Frequently touched hospital surfaces that are more likely contacted mutually by patients, visitors, and health care workers, leftover drugs that remained after the patient used it, and leftover 80% ethanol were included in this study. However, Surfaces that were not mutually touched to the activities of health care workers, patients, and visitors, empty leftover drugs, and absence of leftover 80% ethanol at the petridish on which the alcohol deposited were excluded.

### Sample size determination and sampling techniques

The sample size was determined using a single population proportion formula. Since there are no study reports of bacterial isolates on high touch surfaces, leftover drugs, and 80% ethanol in the study area and no pilot study performed in the study area, the sample size was determined using the expected prevalence of 50%, precision level 5% and confidence interval 95%. Therefore, a total of 384 samples, 136 from high touch surfaces, 208 from leftover drugs, and 40 from 80% ethanol were collected using a convenient sampling technique.

### Sample collection

Environmental samples from high-touch surfaces such as ward sinks, door handles, patients’ bed sheets, bedside tabletops, and blood withdrawal tables were collected at the time the patient and health care workers occupied the room. Briefly, sterile test tubes and cotton-tipped swabs moistened with sterile normal saline were used to collect surface samples [5]. At each sampling site, sterile cotton swabs moistened with sterile normal saline were used to collect surface samples on 10 cm by 10 cm area/100 cm^2^/ surfaces [11]. Surface samples were collected every morning after the cleaning was completed [33]. Moreover, leftover drugs were collected immediately after the medication was given to the patient by the nurse. Before sampling, the medication vials were shaken vigorously and the top cover rubber was disinfected with 705 ethanol. The cover rubber of each vial was opened by using sterile forceps and the Leftover content of the vial was sampled using sterile cotton tipped swab.

According to the WHO alcohol-based handrub formulations, 80% ethanol was prepared by mixing 833.3 ml of ethanol (96%), 41.7 ml of H_2_O_2_ (3%) and 14.5 ml of glycerol (98%) dissolved in 1000 ml of distilled water. Twenty-five ml of 80% ethanol was poured aseptically in to the 150 mm sterile petridish containing 10 small sized sterile cotton swabs, and distributed for each of the hospital wards. All the cotton swabs soaked in the 80% ethanol were used for disinfection purposes by the health worker at each of the hospital wards but swab samples were collected from the leftover alcohol in the petridish. Then, a sterile swab was used to take the ethanol sample left in the petridish, followed by depositing it in sterile tryptone soy broth. All type of samples were labeled properly with a unique identification number at the time of collection and transported using vaccine carrier which had ice box to maintain the temperature at 2-8 °C until it reaches to bacteriology laboratory of UoGCSH.

### Laboratory identification techniques

The collected surface swab samples were inoculated onto Blood agar, MacConkey agar, Chocolate agar and Mannitol salt agar and incubated at 37 °C for 24–48 h. Furthermore, swab samples collected from leftover drugs and 80% ethanol pre-incubated at 37 °C for 24 h to enhance bacteria multiplication in broth before inoculation to solid culture media. Then, broths were centrifuged at 3000 rpm for 3 min and finally the sediment inoculated on to Blood agar, MacConkey agar, Chocolate agar and Mannitol salt agar and incubated aerobically at 37 °C for 24–48 h. Gram-negative bacteria were identified using a series of biochemical tests such as Simmons citrate agar, indole production, urease test, lysine decarboxylase, oxidase test, triple sugar iron agar, H_2_S production, citrate utilization and motility tests. On the other hand, Gram-positive bacteria were identified based on Gram reaction, hemolytic pattern, optochin test, bacitracin test, catalase, coagulase, bile esculin, and salt tolerance tests [33].

### Antimicrobial susceptibility test

Antimicrobial susceptibility test was carried out for each bacterial isolate using Kirby–Bauer disc diffusion method. Briefly, three to five selected pure colonies were taken and transferred to a tube containing 5 ml of sterile normal saline and mixed gently to form a homogeneous suspension until the turbidity of the suspension becomes adjusted to 0.5 McFarland standards [34]. Then, using sterile cotton-tipped swabs, the bacteria distribute evenly over the entire surface of Mueller-Hinton agar (MHA), and MHA supplemented with 5% sheep blood used for *Streptococcus* and *Enterococcus* species. The inoculated plates were left at room temperature for 15 min, and then using sterile forceps a set of antibiotic discs were placed on the inoculated MHA plates.

Antimicrobials were selected according to Clinical Laboratory Standard Institute guideline (CLSI 2021) and these antibiotic discs were ciprofloxacin (5 μg), gentamicin (10 μg), tetracycline (30 μg), cotrimoxazole (25 μg), ceftazidime (30 μg), vancomycin (30 μg), piperacillin (100 μg), imipenem (10 μg), meropenem (30 μg), cefuroxime (30 μg), amikacin (30 μg), cefepime (30 μg), augmentin (30 μg), ampicillin (10 μg), penicillin (10 IU), erythromycin (15 μg), cefoxitin (30 μg), doxycycline (30 μg), and clindamycin (2 μg) [30]. After placing these antibiotic discs, the plates were allowed to stand for another 15 min at room temperature to dissolve antibiotics in the media. The plates then were incubated at 37oC for 18 to 24 h. Finally, zones of inhibitions were measured using a ruler and interpreted according CLSI 2021guidelines [35]. Cefoxitin susceptibility of *S. aureus* and CoNS isolates were tested by placing cefoxitin (30 μg) antibiotic discs on MHA using Kirby–Bauer disc diffusion method and then incubated aerobically at 35^∘^C for 24 h. Finally, *S. aureus* isolates zone of inhibition > 22 mm classified as susceptible and < 21 as non-susceptible. *S. aureus* isolates resistant to cefoxitin (≤ 21 mm) were confirmed as methicillin-resistant *S. aureus*. Furthermore, CoNS isolates zone of inhibition > 25 mm classified as susceptible and < 24 mm as non-susceptible.

### Data quality control and analysis

Sample collection, transportation, bacteriological cultivation, and biochemical assays were done according to the standard operating procedures of the College of Medicine and Health Sciences bacteriology laboratory. Five percent (5%) of the prepared culture media were randomly selected and incubated aerobically for 24 h at 37 °C to cheek the sterility of culture media. In addition, known strains of *S. aureus* (ATCC 25923) and *E. coli* (ATCC 25922) were inoculated to check the performance of the prepared culture media. Inoculation of culture media, colony characterization, and measurement of susceptibility test were checked by an experienced microbiologist at UoGCSH. To standardize the density of the inoculum of bacterial suspension, 0.5 McFarland turbidity standard was used [34]. All data were checked for completeness and analyzed using Statistical Package for Social Sciences software version 20. Descriptive statistics were computed and results were summarized using tables and graphs.

## Results

### Overall culture results

**Fig. 1:**
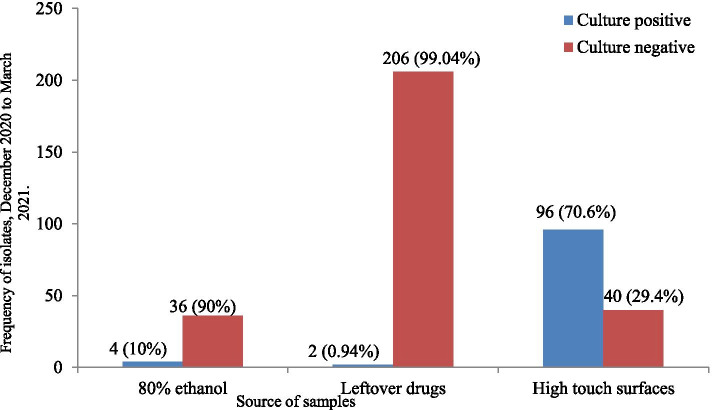
Proportion of bacterial isolates from 80% ethanol, leftover drugs and high touch surfaces from December 2020 to March 2021

In this study, a total of 384 samples were collected and the proportions of samples were 35.4% (*n* = 136), 54.2% (*n* = 208) and 10.4% (*n* = 40) for high touch surfaces, leftover drugs and 80% ethanol, respectively. These samples were from the surgical ward, post-natal ward, orthopedic ward, trauma ward, and neonatal intensive care unit (NICU). Among these swab samples, 26.6% (*n* = 102) were culture positive, and a total of 114 bacteria were isolated. Multi-bacterial contamination was detected in 10.5% (*n* = 12) samples. In this study, a high proportion of bacterial isolates (84.2%; *n* = 96/114) were from high-touch surfaces compared to leftover drugs and 80% ethanol (Fig. 1).

### Frequency of bacterial isolates

Data on the frequency of bacterial isolates showed that 64.9% (*n* = 74) were Gram-positive bacteria but the rest 35.1% (*n* = 40) were Gram-negative bacteria. Among the Gram-positive bacteria isolates, the proportion of coagulase negative *Staphylococci* (CoNS), *S. aureus* and *Enterococcus* species were 59.5% (*n* = 44), 20.3% (*n* = 15) and 12.2% (*n* = 9), respectively. On the other hand, the proportion of Gram-negative bacteria isolates were 32.5% (*n* = 13), 25% (*n* = 10), 17.5% (*n* = 7), and 10% (*n* = 4) for *P. aeruginosa*, *K. pneumoniae*, *A. baumannii* and *E. cloacae*, respectively (Table 1).

**Table 1:** Frequency of Gram-positive and Gram-negative bacteria from December 2020 to March 2021

	Types of species	Frequency (N)	Percent (%)
**Bacterial Isolates**	CoNS	44	38.6%
	*Staphylococcus aureus*	15	13.2%
	*Enterococcus* species	9	7.9%
	Viridans streptococci	3	2.63%
	*Streptococcus pyogenes*	2	1.75%
	*Bacillus* species	1	0.88%
	*Pseudomonas aeruginosa*	13	11.4%
	*Klebsiella pneumoniae*	10	8.8%
	*Acinetobacter baumannii*	9	7.9%
	*Enterobacter cloacae*	4	3.51%
	*Providentia stuartii*	2	1.75%
	*Klebsiella ozaenae*	1	0.88%
	*Burkholderia cepacia*	1	0.88%
	**Total**	**114**	**100**

*CoNS* coagulase negative Staphylococci

### Proportion of bacterial isolates among different hospital wards

In the current study, the highest bacterial isolates were detected from surgical ward (24.6%; *n* = 28), followed by post-natal ward (21%; *n* = 24), orthopedic ward (20.2%; *n* = 23), trauma ward (18.4%; *n* = 21), and NICU (15.8%; *n* = 18). The frequency of Gram-positive bacteria was 57% in the surgical ward, 66.7% in the post-natal ward, 78.3% in the orthopedic ward, 66.7% in the trauma ward, and 55.6% in NICU. The predominant Gram-positive bacteria were CoNS responsible for 42.9% in the surgical ward, 56.5% in the orthopedic ward, 42.9% in the trauma ward, and 33.3% in NICU. *S. aureus* was the dominant bacteria isolated in the post-natal ward accounting for 20.8%. On the other hand, the proportion of the Gram-negative bacteria was 30% in the surgical ward, 20% in each post-natal ward and NICU, 17.5% in the trauma ward, and 12.5% in the orthopedic ward. Among the Gram-negative bacteria isolates, *P. aeruginosa* was the dominant bacteria (39.3%) in the surgical ward, *K. pneumoniae* in the post-natal ward (12.5%), and in NICU (27.8%). Moreover, *A. baumannii* accounted for 14.3 and 8.7% proportion among trauma and orthopedic wards, respectively (Table 2).

**Table 2:** The proportion of bacteria isolated from different wards at UoGCSH from December 2020 to March 2021

Bacterial isolates	Type of ward					
	Surgical n (%)	Post-natal n (%)	Orthopedic n (%)	Trauma n (%)	NICU n (%)	Total
CoNS	12 (42.9)	4 (16.7)	13 (56.5)	9 (42.9)	6 (33.3)	44 (38.6)
*Staphylococcus aureus*	3 (10.7)	5 (20.8)	4 (17.4)	2 (9.5)	1 (5.6)	15 (13.2)
*Enterococcus* species	1 (3.6)	4 (16.7)	–	2 (9.5)	2 (11.1)	9 (7.8)
Viridans streptococci	–	1 (4.2)	1 (4.3)	1 (4.8)	–	3 (2.6)
*Streptococcus pyogenes*	–	1 (4.2)	–	–	1 (5.6)	2 (1.8)
*Bacillus* species	–	1 (4.2)	–	–	–	1 (0.9)
*Pseudomonas aeruginosa*	11 (39.3)	2 (8.3)	–	–	–	13 (11.4)
*Klebsiella pneumoniae*	–	3 (12.5)	–	2 (9.5)	5 (27.8)	10 (8.8)
*Acinetobacter baumannii*	1 (3.6)	1 (4.2)	2 (8.7)	3 (14.3)	2 (11.1)	9 (7.8)
*Enterobacter cloacae*	–	1 (4.2)	1 (4.3)	1 (4.8)	1 (5.6)	4 (3.5)
*Providentia stuartii*	–	–	2 (8.7)	–	–	2 (1.8)
*Klebsiella ozaenae*	–	–	–	1 (4.8)	–	1 (0.9)
*Burkholderia cepacia*	–	1 (4.2)	–	–	–	1 (0.9)
**Total**	**28 (24.6)**	**24 (21)**	**23 (20.2)**	**21 (18.4)**	**18 (15.8)**	**114**

*CoNS* coagulase negative Staphylococci, *NICU* Neonatal Intensive Care Unit

### The proportion of bacteria isolates from different type of samples

Data were collected from 136 high touch surfaces such as bedsheet (*n* = 36), bedside table (*n* = 37), sink (*n* = 25), door handles (*n* = 24) and blood withdrawal tables (*n* = 14) (Table 3). All samples collected from bedside tables and bedsheets were positive for bacterial contamination. Among the bacteria isolated, the proportion of CoNS was 31.8% for bedside tables, 43.2% for bedsheets, and 11.4% for door handles. Sinks contaminated with *Klebsiella* species (81.8%) and *A. baumannii* (55.6%). Blood withdrawal tables were mainly contaminated by *Enterococcus* species (22.2%). On the other hand, *A. baumannii* (22.2%) was the most contaminant for ethanol-based hand rub formulations. Among the leftover drugs, only one vial was contaminated by CoNS and the other one by *S. pyogenes* (Table 3). These contaminated leftover drugs were lidocaine and adrenalin injection from the trauma ward and ceftriaxone from the post-natal ward. All of the leftover drugs collected from surgical ward (*n* = 45), orthopedic ward (*n* = 45) and NICU (*n* = 35) were negative for bacterial contamination. Among 40 samples of 80% ethanol, four samples showed bacterial growth. Three of them were from the orthopedic ward and one from the surgical ward. However, ethanol-based hand rub formulations collected from trauma, post-natal ward, and NICU were negative for bacterial contamination.

**Table 3:** The proportion of bacterial isolates at UoGCSH from December 2020 to March 2021

Isolated bacteria, n (%)												
Sampling points	CoNS	***S. aureus***	***Enterococcus*** species	***Streptococcus*** species	***Bacillus*** species	***P. aeruginosa***	***Klebsiella*** species	***A. baumannii***	***E. cloacae***	***P. stuartii***	***B. cepacia***	Total n (%)
Bed sheet	19 (43.2)	6 (40)	2 (22.2)	0 (0)	0 (0)	5 (38.5)	1 (9.1)	2 (22.2)	0 (0)	0 (0)	1 (100)	36 (31.6)
Bed side table	14 (31.8)	8 (53.3)	5 (55.6)	1(20)	0 (0)	5 (38.5)	1 (9.1)	0	3 (75)	0 (0)	0 (0)	37 (32.5)
Sink	3 (6.8)	0 (0)	0 (0)	2 (40)	0 (0)	3 (23.1)	9 (81.8)	5 (55.6)	1 (25)	2 (100)	0 (0)	25 (21.9)
Door handles	5 (11.4)	0 (0)	0 (0)	1 (20)	0 (0)	0 (0)	0 (0)	0 (0)	0 (0)	0 (0)	0 (0)	6 (5.3)
Blood culture table	1 (2.3)	1 (6.7)	2 (22.2)	0 (0)	0 (0)	0 (0)	0 (0)	0 (0)	0 (0)	0 (0)	0 (0)	4 (3.5)
80% ethanol	1 (2.3)	0 (0)	0 (0)	0 (0)	1 (100)	0 (0)	0 (0)	2 (22.2)	0 (0)	0 (0)	0 (0)	4 (3.5)
Leftover drugs	1 (2.3)	0 (0)	0 (0)	1 (20)	0	0 (0)	0 (0)	0 (0)	0 (0)	0 (0)	0 (0)	2 (1.8)
**Total**	**44**	**15**	**9**	**5**	**1**	**13**	**11**	**9**	**4**	**2**	**1**	**114**

*CoNS* coagulase negative Staphylococci

### Antimicrobial susceptibility profile of gram-positive bacteria isolates

A total of 73 Gram-positive bacteria were subjected to antimicrobial susceptibility test and the majority of the CoNS isolates were susceptible to gentamicin (65.9%, *n* = 29), clindamycin (63.6%, *n* = 28) and doxycycline (63.6%, *n* = 28). Half of the CoNS isolates were susceptible to ciprofloxacin (50%, *n* = 22), and tetracycline (50%, *n* = 22). However, only lower number of CoNS isolates were susceptible to erythromycin (38.6%; *n* = 17), penicillin (31.8%; *n* = 14) and cotrimoxazole (21.7%; *n* = 10). The majority of *S. aureus* isolates were susceptible to gentamicin and cotrimoxazole (each 53.3%; *n* = 8) and ciprofloxacin (60%; *n* = 9). Additionally, the majority of the *Enterococci* species were susceptible to ampicillin (77.8%, *n* = 7), gentamicin (66.7%, *n* = 6) and vancomycin (66.7%, *n* = 6). Moreover, one *Viridans streptococci* isolate was susceptible to erythromycin, clindamycin, tetracycline, cefepime, and vancomycin. All *S. pyogenes* isolates were found susceptible to erythromycin but non-susceptible to other tested antibiotics. On the other hand, 50% (*n* = 22) of the CoNS isolates were non-susceptible to cefoxitin (< 24), which is considered methicillin-resistant CoNS. While 13.3% (*n* = 2) of *S. aureus* isolates were susceptible to cefoxitin (> 22) and 86.7% (*n* = 13) of *S. aureus* isolates were non-susceptible to cefoxitin (< 21), which confirmed as methicillin-resistant *S. aureus*. Gram-positive bacteria had significantly high resistance levels to penicillin (67.6%), cotrimoxazole (67.8%), and cefepime (80%) (Table 4).

**Table 4:** AST of Gram-positive bacteria at UoGCSH from December 2020 to March 2021

Bacterial isolates (n)		Antimicrobial agents											
	Pattern	ERY	CD	GEN	P	CXT	TE	DOX	CIP	COT	CFP	VAN	AMP
*CoNS* (44)	*S*	17 (38.6)	28 (63.6)	29 (65.9)	14 (31.8)	22 (50)	22 (50)	28 (63.6)	22 (50)	10 (22.7)	ND	ND	ND
	*NS*	27 (61.4)	16 (36.4)	15 (34.1)	30 (68.2)	22 (50)	22 (50)	16 (36.4)	22 (50)	34 (77.3)	ND	ND	ND
*S. aureus* (15)	*S*	7 (46.7)	7 (46.7)	8 (53.3)	4 (26.7)	2 (13.3)	6 (40)	6 (40)	9 (60)	8 (53.3)	ND	ND	ND
	*NS*	8 (53.3)	8 (53.3)	7 (46.7)	11(73.3)	13 (86.7)	9 (60)	9 (60)	6 (40)	7 (46.7)	ND	ND	ND
*Enterococcus* species (9)	*S*	ND	ND	6 (66.7)	4 (44.4)	ND	ND	ND	ND	ND	ND	6 (66.7)	7 (77.8)
	*NS*	ND	ND	3 (33.3)	5 (55.6)	ND	ND	ND	ND	ND	ND	3 (33.3)	2 (22.2)
*Viridans streptococci* (3)	*S*	1 (33.3)	1 (33.3)	ND	ND	ND	1 (33.3)	ND	ND	ND	1 (33.3)	1 (33.3)	ND
	*NS*	2 (66.7)	2 (66.7)	ND	ND	ND	2 (66.7)	ND	ND	ND	2 (66.7)	2 (66.7)	ND
*S. pyogenes (2)*	*S*	2 (100)	0	ND	0	ND	0	ND	ND	ND	0	0	ND
	*NS*	0	2 (100)	ND	2 (100)	ND	2 (100)	ND	ND	ND	2 (100)	2 (100)	ND
Total (73)	*S*	27 (42.2)	36 (56.3)	43 (63.2)	22 (32.4)	24 (40.7)	29 (46.8)	34 (57.6)	31 (52.5)	18 (30.5)	1 (20)	ND	7 (77.8)
	*NS*	37 (57.8)	28 (43.7)	25 (36.8)	46 (67.6)	35 (59.3)	33 (53.2)	25 (42.4)	28 (47.5)	41 (67.8)	4 (80)	ND	2 (22.2)

*AST* antimicrobial susceptibility test, *CoNS* coagulase negative Staphylococci, *ERY* erythromycin, *CD* clindamycin, *GEN* gentamicin, *PEN* penicillin, *CXT* cefoxitin, *TE* tetracycline, *DOX* doxycycline, *CIP* ciprofloxacin, *COT* cotrimoxazole, *CFP* cefepime, *VAN* vancomycin, *AMP* ampicillin, *ND* susceptibility test not done, *S* susceptible, *NS* non-susceptible

### Antimicrobial susceptibility pattern of the gram-negative bacteria isolates

In this study, antimicrobial susceptibility tests were done for 40 Gram-negative bacteria isolates. All isolates of *P. aeruginosa* (100%, n = 13) isolates were susceptible to gentamicin, while eleven (84.6%) and nine (69.2%) of them were susceptible to amikacin and imipenem, respectively. But only one *P. aeruginosa* isolate was susceptible to ciprofloxacin and piperacillin. The majority of *A. baumannii* isolates were susceptible to ciprofloxacin (88.9%), amikacin (88.9%), and 77.8% of them were susceptible to cotrimoxazole and imipenem. Data also demonstrated that all *K. ozaenae* isolates were susceptible to all tested antimicrobial agents, except augmentin. Furthermore, *K. pneumoniae* isolates were susceptible to amikacin, gentamicin, cefepime, cotrimoxazole, imipenem, ceftazidime, cefuroxime and ciprofloxacin; (9, 90%), (70%, 7), (70%, 7), (70%, 7), (70%, 7), (60%, 6), (50%, 5), and (50%, 5), respectively. Amikacin (100%), imipenem (100%), ciprofloxacin (75%), cefuroxime (50%), gentamicin (50%), and ceftazidime (50%) are effective against *E. cloacae* isolates. Moreover, amikacin (100%), gentamicin (100%), and imipenem (100%) are effective antimicrobial agents for *P. stuartii* isolates, and meropenem (100%) effective for *B. cepacia* isolate. Gram-negative bacteria revealed the highest resistance levels to tetracycline (82.4%), amoxicillin-clavulanic acid (76.5%), cefepime (66.7%), ceftazidime (67.5%), and piperacillin (92.3%) (Table 5).

**Table 5:** AST of Gram-negative bacteria at UoGCSH from December 2020 to March 2021

Antimicrobial agents													
Bacterial isolates (n)	Sensitivity pattern	TE	CIP	AMC	AN	CXM	GEN	CFP	COT	IPM	CAZ	PRL	MPN
*P. aeruginosa* (13)	*S*	ND	1 (7.7)	ND	11 (84.6)	ND	13 (100)	0	ND	9 (69.2)	0	1 (7.7)	ND
	*NS*	ND	12 (92.3)	ND	2 (15.4)	ND	0	13 (100)	ND	4 (23.1)	13 (100)	12 (92.3)	ND
*A. baumannii (9)*	*S*	ND	8 (88.9)	ND	8 (88.9)	ND	5 (55.6)	3 (33.3)	7 (77.8)	7 (77.8)	4 (44.4)	ND	ND
	*NS*	ND	1 (11.1)	ND	1 (11.1)	ND	4 (44.4)	6 (66.7)	2 (22.2)	2 (22.2)	5 (55.6)	ND	ND
*K. pneumoniae* (10)	*S*	2 (20)	5 (50)	2 (20)	9 (90)	5 (50)	7 (70)	7 (70)	7 (70)	7 (70)	6 (60)	ND	ND
	*NS*	8 (80)	5 (50)	8 (50)	1 (10)	5 (50)	3 (30)	3 (30)	3 (30)	3 (30)	4 (40)	ND	ND
*E. cloacae* (4)	*S*	0	3 (75)	1 (25)	4 (100)	2 (50)	2 (50)	1 (25)	1 (25)	4 (100)	2 (50)	ND	ND
	*NS*	4 (100)	1 (25)	3 (75)	0	2 (50)	2 (50)	3 (50)	3 (75)	0	2 (50)	ND	ND
*P. stuartii* (2)	*S*	0	0	1 (50)	2 (100)	0	2 (100)	1 (50)	0	2 (100)	0	ND	ND
	*NS*	2 (100)	2 (100)	1 (50)	0	2 (100)	0	1 (50)	2 (100)	0	2 (100)	ND	ND
*K. ozaenae* (1)	*S*	1 (100)	1 (100)	0	1 (100)	1 (100)	1 (100)	1 (100)	1 (100)	1 (100)	1 (100)	ND	ND
	*NS*	0	0	1 (100)	0	0	0	0	0	0	0	ND	ND
*B. cepacia* (1)	*S*	ND	ND	ND	ND	ND	ND	ND	0	ND	0	ND	1 (100)
	*NS*	ND	ND	ND	ND	ND	ND	ND	1 (100)	ND	1 (100)	ND	0
Total (40)	*S*	3 (17.6)	18 (46.2)	4 (23.5)	35 (89.7)	8 (47)	31 (79.5)	13 (33.3)	16 (94.1)	30 (76.9)	13 (32.5)	1 (7.7)	1 (100)
	*NS*	14 (82.4)	21 (53.8)	13 (76.5)	4 (10.3)	9 (53)	8 (20.5)	26 (66.7)	11 (5.9)	9 (23.1)	27 (67.5)	12 (92.3)	0

*AST* antimicrobial susceptibility test, *TE* tetracycline, *CIP* ciprofloxacin, *AMC* amoxicillin clavulanic acid, *AN* amikacin, *CXM* cefuroxime, *GEN* gentamicin, *CFP* cefepime, *COT* cotrimoxazole, *IPM* imipenem, *CAZ* ceftazidime, *PRL* piperacillin, *MPN* meropenem, *ND* susceptibility test not done, *S* susceptible, *NS* non-susceptible

### Multidrug resistance pattern of bacterial isolates

Multidrug-resistant isolates refer to an isolate resistant to at least one antibiotic in three or more drug classes. In this study, the prevalence of MDR isolates was 44.7% (*n* = 51). Multidrug-resistance was detected among Gram-positive and Gram-negative bacteria, 54.8 and 27.5%, respectively. Out of 114 bacterial isolates, 59.l % (*n* = 26) of the CoNS isolates were found multidrug resistant and 36.4% (*n* = 16) were methicillin-resistant. Ten *S. aureus* isolates (66.7%) were multidrug-resistant, and 13 of the *S. aureus* (86.7%) isolates were MRSA. All *S. pyogenes* and *P. stuartii* isolates were found MDR. Surprisingly, two *S. aureus* isolates from bedsheet and two *viridian streptococci* isolated from the sink were non-susceptible to all tested antimicrobials (Table 6).

**Table 6:** MDR pattern of bacterial isolates at UoGCSH from December 2020 to March 2021

Bacterial isolates	Number of MDR isolates (%)	Antimicrobials resisted by most isolates
CoNS	26/44 (59.1)	ERY, PEN, COT
*Staphylococcus aureus*	10/15 (66.7)	CD, PEN, CXT, TE, DOX
*Viridans streptococci*	2/3 (66.7)	ERY, CD, TE, CFP, VAN
*Streptococcus pyogenes*	2/2 (100)	PEN, CD, CFP, TE, VAN
*Pseudomonas aeruginosa*	4/13 (30.8)	CIP, CAZ, CFP, PRL
*Klebsiella pneumoniae*	3/10 (30)	TE, CIP, AMC, CXM
*Enterobacter cloacae*	2/4 (50)	AMC, TE, COT
*Providentia stuartii*	2/2 (100)	CAZ, CXM, COT, CIP, TE

*MDR* multi-drug resistant, *CoNS* coagulase negative Staphylococci, *ERY* erythromycin, *PEN* penicillin, *COT* cotrimoxazole, *CD* clindamycin, *CXT* cefoxitin, *TE* tetracycline, *DOX* doxycycline, *CFP* cefepime, *VAN* vancomycin, *CIP* ciprofloxacin, *CIP* ciprofloxacin, *CAZ* ceftazidime, *PRL* piperacillin, *AMC* amoxicillin clavulanic acid, *CXM* cefuroxime

## Discussion

High-touch surfaces in the hospital are potential sources of nosocomial infections, which increases the risk of contamination among susceptible hosts [36, 37]. In this study, the overall bacterial contaminations of high touch surfaces, leftover drugs, and 80% ethanol was 26.6%, which is lower than a study carried out at Tikur Anbessa Specialized Teaching Hospital, Addis Ababa, Ethiopia (86%) [38]. This difference might be due to differences in diagnostic techniques and infection control policies. Data showed that CoNS and *S. pyogenes* were isolated from both high-touch surfaces and leftover drugs. On the other hand, CoNS and *A. baumannii* were isolated from both high-touch surfaces and 80% ethanol. The possible reason might be the existence of bacterial cross-contamination among high-touch surfaces, leftover drugs, and 80% ethanol.

In this study, culture result showed that 70.6% of high-touch surfaces were contaminated by bacteria. Bacterial contamination of high-touch surfaces reported in Zimbabwe [39] and Morocco [40] were reported 86.2 and 96.3%, respectively. On the other hand, previous study reports from Ethiopia [11], Sudan [41], and Nigeria [42] demonstrated that bacterial contamination rate were 46.3, 29.7, and 39.4%, respectively. Differences in hand hygiene, frequency of surfaces decontamination, ventilation system, the use of antiseptics, and disinfection techniques could account for these discrepancies. Higher levels of bacterial contamination of high touch surfaces observed in this study could be attributed primarily due to the use of ineffective disinfectants during surface cleaning, inadequate use of standard precautions (hand hygiene and contact precautions), and the migration of the organisms through airflow [26, 39, 43]. This situation is prominently linked to hospitals that show an unwillingness to put funds into contamination control such as the ventilation systems, lack of information about the level of contamination and ineffectiveness of commonly used disinfectants in hospitals, and those with inappropriate waste controls [44, 45].

Among the leftover drugs processed, 0.96% (2/208) was found contaminated by CoNS and *S. pyogenes*, which entailed that the drugs that were administered to patients might be contaminated by bacteria from the hands of attendants that brought those drugs from the pharmacy to the ward, contaminated air, and surfaces of the ward. Previously, Baniasadi S. et al reported on microbial contamination of single and multiple-dose vials (5.36%) [14]. In contrast, another study conducted in Iran on bacterial contamination of single and multiple-dose vials after multiple uses revealed that none of the vials were contaminated [46]. This difference could be due to the difference in sanitation practices that have been established like contamination of nurses’ hands/room, air, and surfaces of the treatment room.

From the total 80% ethanol samples processed, 4 (10%) showed bacterial growth. This finding was higher than the studies conducted in Thailand (1.8%) [18]. This difference could be due to the difference in sample size, hygienic status of the hospital environment, and the way of using alcohol by health care personnel. Microorganisms isolated from 80% ethanol were mainly environmental microbes. *Bacillus* species, CoNS, and *A. baumannii* were the isolated bacteria, and the highest number of isolates recovered in the orthopedic ward followed by the surgical ward. This implies that the risk of contracting the nosocomial infection from 80% ethanol in orthopedic ward might be higher. The highest number of isolates in the orthopedic ward could be due to incorrect aseptic technique followed by the nurse while using the 80% ethanol.

In the present study, 64.9 and 34.1% of isolates were Gram-positive and Gram-negative, respectively. The dominancy of Gram-positive bacteria was reported in studies in Bahirdar (81.6%) [11], Tikur Anbesa hospital (56.3%) [12], Mekelle (68.4%) [47], and in abroad in Iran (60.7%) [48] and Nigeria (52.2%) [49]. The higher frequency of Gram-positive bacteria might be due to resistance to dry conditions of the hospital environment and transmission from the skin and nasal cavity of health care personnel and patients. However, studies conducted in Zimbabwe [39] and Morocco [40] showed Gram-negative bacteria as the predominant environmental isolates. These variations could be due to differences in the study period, hospital setting, and the visit of already colonized or infected patients in the ward.

Among different wards examined, the highest numbers of bacteria were recovered from the surgical ward (24.6%), followed by post-natal (21%) and orthopedic ward (20.2%). This finding is in line with a study conducted from the surgical ward, the post-natal and orthopedic wards in Bahir Dar were 32.6, 25.9, 16.3%, respectively [11]. The possible explanation for the reported high bacterial contamination in these wards could be due to high and unrestricted human trafficking, particularly medical and health science students who were attached to the hospital as part of their practical learning process and visitors of the patients. The highest bacterial contaminated samples were from the bedside tables and bedsheets, which is in line with the findings in Ethiopia [11] and Morocco [50]. Bedside tables and bedsheets were mainly contaminated by CoNS (31.8 and 43.2%), *S. aureus* (53.3 and 40%), and *P. aeruginosa* (38.5% each), respectively. Comparable results were obtained on bedside tables and bed samples from studies conducted in Morocco [50]. The sources of such contamination could be cross-contamination from a patient’s flora, health care workers’ hands, and contamination during the washing process.

On the other hand, CoNS isolates were the most frequently isolated bacteria, 44 (38.6%) followed by *S. aureus*, 15 (13.2%), which is Lower than the findings from Bahirdar referral Hospital (44 and 37.4%) [5] but relatively higher than the report from Ayder Comprehensive Specialized Hospital (35.4 and 29.1%) [47], northern Ethiopia. *S. aureus* constitutes the normal human skin and mucous membranes flora [51], and they are regularly shed onto the hospital environment by patients and medical personnel. These isolates were also indicators of inadequate clinical surface hygiene [52, 53]. Moreover, these bacteria were also resistant to common disinfectant methods and hence spread easily in the environment, which enables them to colonize and infect the patients receiving health care services [54].

The majority of the isolates of CoNS showed susceptibility to gentamicin (65.9%), clindamycin (63.6%), and doxycycline (63.6%). This result agrees with a study conducted in Bahir Dar referral hospital (73.3, 82.8 and 71%) [11] and Tikur Anbesa (86.4, 96.3 and 55.7%), respectively [12]. On the other hand, majority of *S. aureus* isolates were susceptible to gentamicin (53.3%), ciprofloxacin (60%) and cotrimoxazole (53.3%). This finding agrees with the study reported in Bahir Dar (73.3, 77.5 and 50.5%) [11] and Tikur Anbesa (90.4, 89.2 and 65.8%) [12], respectively. The majority of the *Enterococcus* species showed susceptibility to vancomycin (66.7%) and ampicillin (77.8%). This result agrees with the report in Tikur Anbesa [12] with a sensitivity of 75% to ampicillin. The majority of *P. aeruginosa* isolates were susceptible to amikacin (84.6%), gentamicin (100%), and imipenem (69.2%), which agrees with a study reported from Tikur Anbesa; amikacin (100%), and gentamicin (87.5%) [12]. The isolates of *A. baumannii* showed susceptibility to ciprofloxacin (88.9%), amikacin (88.9%), gentamicin (88.9%), cotrimoxazole (77.8%), and imipenem (77.8%), which agrees reports from Tikur Anbesa [12].

Most of the *K. pneumoniae* isolates showed susceptibility to amikacin (90%), 70% each to gentamicin, cotrimoxazole, cefepime, imipenem, and 60% to ceftazidime, which agrees with the study reported from Bahir Dar [11] with their susceptibility result of gentamicin (59%) and cotrimoxazole (73%) and Tikur Anbesa [12] with their susceptibility result of gentamicin (62.5%) and ciprofloxacin (75%). The majority of *E. cloacae* showed sensitivity to ciprofloxacin (75%), amikacin (100%), and imipenem (100%). This finding agrees with the study reported by Tikur Anbesa [12] with 100% susceptibility to ciprofloxacin. The findings of this study showed that 44.7% of bacterial isolates were multidrug-resistant. This finding is lower than reports from Bahirdar (75%) [11], Tikur Anbesa (56%) [12], Zimbabwe (75%) [39] and Iran (79.4%) [48]. Multi-drug resistance was detected among Gram-positive and Gram-negative bacteria, 54.8 and 27.5%, respectively. This finding disagrees with the reports from Tikur Anbesa (45.8 and 73.8%) [12] and Iran (47.8 and 83.1%) [48], respectively. The persistent pressure of disinfectants on the microorganisms present in the hospital environment may lead to the emergence of MDR strains [55]. As a limitation of this study, selection bias might be introduced because of the convenient sampling technique. Additionally, we didn’t address risk factors in this study.

## Conclusion and recommendations

In this study, different types of bacterial isolates were identified at different settings of the hospital that could predispose patients attending medical care. CoNS, *S. aureus,* and *P. aeruginosa* were the most commonly isolated bacteria from high-touch surfaces. Significant multidrug-resistance isolates were found from high-touch surfaces and the vial of ceftriaxone. Multidrug-resistance isolates were found among CoNS and *S. aureus* bacteria isolates, and a significant proportion of the *S. aureus* isolates were methicillin resistant. Regular sanitation and disinfection, continuous surveillance and monitoring of the bacterial types and their drug susceptibility patterns from contact surfaces and antiseptics should be practiced periodically to minimize the cross-contamination of bacteria to medications and antiseptics.

## Data Availability

All data generated or analyzed during this study were included in this article. Data that support the findings of this study are also available from the corresponding author upon reasonable request.

## Abbreviations

CLSI: Clinical Laboratory Standards Institute
MHA: Muller Hinton Agar
MDR: Multidrug-Resistant
NICU: Neonatal Intensive Care Unit
NIs: Nosocomial Infections
UoGCSH: University of Gondar Comprehensive Specialized Hospital.

## References

[1] Ducel G, Fabry J, Nicolle L (2002). Prevention of hospital acquired infections: a practical guide.

[2] Klevens RM, Edwards JR, Richards CL, Horan TC, Gaynes RP, Pollock DA (2007). Estimating health care-associated infections and deaths in US hospitals, 2002. Public Health Rep.

[3] Boyce JM (2007). Environmental contamination makes an important contribution to hospital infection. J Hosp Infect.

[4] Huslage K, Rutala WA, Gergen MF, Sickbert-Bennett EE, Weber DJ (2013). Microbial assessment of high, medium, and low touch hospital room surfaces. Infect Control Hosp Epidemiol.

[5] Worku T, Derseh D, Kumalo A. Bacterial profile and antimicrobial susceptibility pattern of the isolates from stethoscope, thermometer, and inanimate surfaces of Mizan-Tepi University Teaching Hospital, Southwest Ethiopia. International journal of microbiology. 2018;2018. 10.1155/2018/9824251 PMC6040268 30050575

[6] Huslage K, Rutala WA, Sickbert-Bennett E, Weber DJ (2010). A quantitative approach to defining “high-touch” surfaces in hospitals. Infect Control Hosp Epidemiol.

[7] Cheng VC, Chau PH, Lee WM, Ho SK, Lee DW, So SY (2015). Hand-touch contact assessment of high-touch and mutual-touch surfaces among healthcare workers, patients, and visitors. J Hosp Infect.

[8] Sun C, Hu YJ, Wang X, Lu J, Lin L, Zhou X (2019). Influence of leftover antibiotics on self-medication with antibiotics for children: a cross-sectional study from three Chinese provinces. BMJ Open.

[9] Soubieux A, Tanguay C, Bussières JF. Review of studies examining microbial contamination of vials used for preparations done with closed-system drug transfer devices. European Journal of Hospital Pharmacy. 2021;28(2):65-70. 10.1136/ejhpharm-2019-001913 PMC7907703 33608432

[10] Kumar VA, Khan S (2015). Defining multidrug resistance in gram-negative bacilli. Indian J Med Res.

[11] Getachew H, Derbie A, Mekonnen D. Surfaces and air bacteriology of selected wards at a referral hospital, Northwest Ethiopia: a cross-sectional study. Int J Microbiol. 2018;2018. 10.1155/2018/6413179 PMC5971317 29861733

[12] Endalafer N (2008). Bacterial nosocomial infections and their antimicrobial susceptibility patterns in surgical wards and surgical intensive care unit of Tikur Anbessa university hospital.

[13] Alemayehu T, Tadesse E, Ayalew S, Nigusse B, Yeshitila B, Amsalu A, Assefa A. High burden of Nosocomial infections caused by multi-drug Re-sistant pathogens in pediatric patients at Hawassa university comprehensive specialized hospital. Ethiopian Med J. 2019;58:45-53.

[14] Baniasadi S, Dorudinia A, Mobarhan M, Gamishan MK, Fahimi F (2013). Microbial contamination of single-and multiple-dose vials after opening in a pulmonary teaching hospital. Braz J Infect Dis.

[15] Larmene-Beld KHM, Frijlink HW, Taxis K (2019). A systematic review and meta-analysis of microbial contamination of parenteral medication prepared in a clinical versus pharmacy environment. Eur J Clin Pharmacol.

[16] Jackson MM (2005). Topical antiseptics in healthcare. Clin Lab Sci.

[17] Weber DJ, Rutala WA, Sickbert-Bennett EE (2007). Outbreaks associated with contaminated antiseptics and disinfectants. Antimicrob Agents Chemother.

[18] Danchaivijitr S, Dhiraputra C, Rongrungruang Y, Srihapol N, Pumsuwan V (2005). Microbial contamination of antiseptics and disinfectants. J Med Assoc Thail.

[19] Chroma M, Kolar M (2010). Genetic methods for detection of antibiotic resistance: focus on extended-spectrum β-lactamases. Biomed Pap Med Fac Univ Palacky Olomouc Czech Repub.

[20] Suvikas-Peltonen E, Hakoinen S, Celikkayalar E, Laaksonen R, Airaksinen M (2017). Incorrect aseptic techniques in medicine preparation and recommendations for safer practices: a systematic review. Eur J Hosp Pharm.

[21] Gajadhar T, Lara A, Sealy P, Adesiyun AA (2003). Microbial contamination of disinfectants and antiseptics in four major hospitals in Trinidad. Rev Panamericana Salud Publ.

[22] Alemu AY, Endalamaw A, Bayih WA (2020). The burden of healthcare-associated infection in Ethiopia: a systematic review and meta-analysis. Trop Med Health.

[23] Solomon FB, Wadilo FW, Arota AA, Abraham YL (2017). Antibiotic resistant airborne bacteria and their multidrug resistance pattern at university teaching referral Hospital in South Ethiopia. Ann Clin Microbiol Antimicrob.

[24] Engda T, Moges F, Gelaw A, Eshete S, Mekonnen F. Prevalence and antimicrobial susceptibility patterns of extended spectrum beta-lactamase producing Enterobacteriaceae in the University of Gondar Referral Hospital environments, Northwest Ethiopia. BMC Res Notes 2018; 11(1):1–7. 10.1186/s13104-018-3443-1 PMC5964971 29788988

[25] Weber DJ, Anderson D, Rutala WA (2013). The role of the surface environment in healthcare-associated infections. Curr Opin Infect Dis.

[26] Santajit S, Indrawattana N. Mechanisms of antimicrobial resistance in ESKAPE pathogens. BioMed research international. 2016;2016. 10.1155/2016/2475067 PMC4871955 27274985

[27] Laudy AE, Róg P, Smolińska-Król K, Ćmiel M, Słoczyńska A, Patzer J (2017). Prevalence of ESBL producing *Pseudomonas aeruginosa* isolates in Warsaw, Poland, detected by various phenotypic and genotypic methods. PLoS One.

[28] Desta K, Woldeamanuel Y, Azazh A, Mohammod H, Desalegn D, Shimelis D (2016). High gastrointestinal colonization rate with extended-spectrum β-lactamase-producing Enterobacteriaceae in hospitalized patients: emergence of carbapenemase-producing *K. pneumoniae* in Ethiopia. PLoS One.

[29] Eshetie S, Tarekegn F, Moges F, Amsalu A, Birhan W, Huruy K (2016). Methicillin resistant *Staphylococcus aureus* in Ethiopia: a meta-analysis. BMC Infect Dis.

[30] Legese MH, Weldearegay GM, Asrat D (2017). Extended-spectrum beta-lactamase-and carbapenemase-producing Enterobacteriaceae among Ethiopian children. Infect Drug Resist.

[31] Ferede ZT, Tullu KD, Derese SG, Yeshanew AG (2018). Prevalence and antimicrobial susceptibility pattern of Enterococcus species isolated from different clinical samples at black lion specialized teaching hospital, Addis Ababa, Ethiopia. BMC Res Notes..

[32] Teklu DS, Negeri AA, Legese MH, Bedada TL, Woldemariam HK, Tullu KD (2019). Extended-spectrum beta-lactamase production and multi-drug resistance among Enterobacteriaceae isolated in Addis Ababa, Ethiopia. Antimicrob Resist Infect Control.

[33] Cheesbrough M (2005). District laboratory practice in tropical countries, part 2.

[34] Hombach M, Maurer FP, Pfiffner T, Böttger EC, Furrer R (2015). Standardization of operator-dependent variables affecting precision and accuracy of the disk diffusion method for antibiotic susceptibility testing. J Clin Microbiol.

[35] Clinical and Laboratory Standards Institute (CLSI). Performance Standards for Antimicrobial Susceptibility Testing. 31st ed. CLSI supplement M100 (ISBN 978-1-68440-104-8 [Print]; ISBN 978-1-68440-105-5 [Electronic]). USA: Clinical and Laboratory Standards Institute; 2021.

[36] Zaib H, Kanwar R, Zafar N, Ali S (2019). Prevalence and multidrug resistance profiles of several bacterial pathogens isolated from hospital inanimate surfaces in Faisalabad, Pakistan. Novel Res Microbiol J.

[37] Mann EE, Manna D, Mettetal MR, May RM, Dannemiller EM, Chung KK (2014). Surface micropattern limits bacterial contamination. Antimicrob Resist Infect Control.

[38] Sebre S, Abegaz WE, Seman A, Awoke T, Desalegn Z, Mihret W (2020). Bacterial profiles and antimicrobial susceptibility pattern of isolates from inanimate hospital environments at Tikur Anbessa specialized teaching hospital, Addis Ababa, Ethiopia. Infect Drug Resist.

[39] Mbanga J, Sibanda A, Rubayah S, Buwerimwe F, Mambodza K. Multi-drug resistant (MDR) bacterial isolates on close contact surfaces and health care workers in intensive care units of a tertiary hospital in Bulawayo, Zimbabwe. J Adv Med Med Res. 2018:1–15.

[40] Lalami AEO, Touijer H, Ettayebi M, Benchemsi N (2016). Microbiological monitoring of environment surfaces in a hospital in Fez city, Morocco surveillance. J Mater Environ Sci.

[41] Nurain AM, Bilal NE, Ibrahim ME (2015). The frequency and antimicrobial resistance patterns of nosocomial pathogens recovered from cancer patients and hospital environments. Asian Pac J Trop Biomed.

[42] Hammuel C, Jatau ED, Whong CM. Prevalence and antibiogram pattern of some nosocomial pathogens isolated from hospital environment in Zaria, Nigeria. Aceh International Journal of Science and Technology. 2014;3(3):131-9.

[43] Weber DJ, Rutala WA (2013). Understanding and preventing transmission of healthcare-associated pathogens due to the contaminated hospital environment. Infect Control Hosp Epidemiol.

[44] Dancer SJ (2014). Controlling hospital-acquired infection: focus on the role of the environment and new technologies for decontamination. Clin Microbiol Rev.

[45] Pittet D, Dharan S, Touveneau S, Sauvan V, Perneger TV (1999). Bacterial contamination of the hands of hospital staff during routine patient care. Arch Intern Med.

[46] Khalili H, Sheikhbabayi M, Samadi N, Jamalifar H, Dalili D, Samadi N (2013). Bacterial contamination of single-and multiple-dose vials after multiple use and intravenous admixtures in three different hospitals in Iran. Iran J Pharmaceut Res.

[47] Darge A, Kahsay AG, Hailekiros H, Niguse S, Abdulkader M. Bacterial contamination and antimicrobial susceptibility patterns of intensive care units medical equipment and inanimate surfaces at Ayder Comprehensive Specialized Hospital, Mekelle, Northern Ethiopia. BMC research notes. 2019;12(1):1-8. 10.1186/s13104-019-4658-5 PMC6757422 31547851

[48] Tajeddin E, Rashidan M, Razaghi M, Javadi SS, Sherafat SJ, Alebouyeh M (2016). The role of the intensive care unit environment and health-care workers in the transmission of bacteria associated with hospital acquired infections. J Infect Public Health.

[49] Maryam A, Hadiza US, Aminu UM (2014). Characterization and determination of antibiotic susceptibility pattern of bacteria isolated from some fomites in a teaching hospital in northern Nigeria. Afr J Microbiol Res.

[50] Bakkali M, Hmid K, Kari K, Zouhdi M, Mzibri M, Lalaoui A (2016). Characterization of bacterial strains and their resistance status in hospital environment. J Trop Dis.

[51] Tong SY, Davis JS, Eichenberger E, Holland TL, Fowler VG (2015). *Staphylococcus aureus* infections: epidemiology, pathophysiology, clinical manifestations, and management. Clin Microbiol Rev.

[52] Dancer SJ (2004). How do we assess hospital cleaning? A proposal for microbiological standards for surface hygiene in hospitals. J Hosp Infect.

[53] Fagade OE, Ezeamagu CO, Oyelade AA, Ogunjobi AA (2010). Comparative study of antibiotic resistance of Staphylococcus species isolated from clinical and environmental samples. AU J Tech.

[54] Rozman U, Turk SŠ. PCR technique for the microbial analysis of inanimate hospital environment. Polymerase Chain Reaction for Biomedical Applications. UK: InTech. 2016';119-34

[55] Chemaly RF, Simmons S, Dale C, Ghantoji SS, Rodriguez M, Gubb J, Stachowiak J, Stibich M (2014). The role of the healthcare environment in the spread of multidrug-resistant organisms: update on current best practices for containment. Therapeut Adv Infect Dis.

